# Unilateral Linear Capillaritis Dermoscopic Examination: A Distinct Clinico-Histopathological Correlation

**DOI:** 10.5826/dpc.1103a53

**Published:** 2021-07-08

**Authors:** Jayanti Singh, Priyadarshini Sahu, Surabhi Dayal, Sant Prakash Kataria

**Affiliations:** 1 Department of Dermatology, Venereology and Leprology, Pt B D Sharma University of Health Sciences, Rohtak, Haryana, India; 2 Department of Pathology, Pt B D Sharma University of Health Sciences, Rohtak, Haryana, India

**Keywords:** Unilateral Linear Capillaritis, Dermoscopy, pigmented purpuric dermatosis

## Introduction

Unilateral linear capillaritis (ULC) is a rare variant of pigmented purpuric dermatosis (PPD). It is characterized by unilateral, progressive, linear eruption of purpuric patches or macules. It is a benign condition and often resolves spontaneously. To the best of our knowledge, ULC dermoscopic features have only been described in 1 case report. Herein, we attempted to compare the dermoscopic features of ULC with PPD and differentiate it from linear pityriasis rosea (PR). In the present case, we also found a few more dermoscopic features characterizing ULC, which have not been reported yet in the literature. To establish ULC dermoscopic features and differentiate it from other linear dermatoses, there is the need to present additional findings to enrich the current documentation.

## Case Report

**Figure 1:**
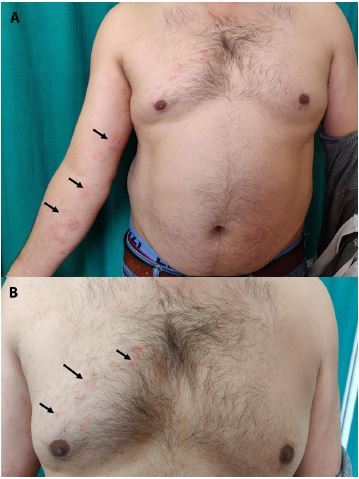
Multiple linear erythematous, slightly scaly, patches seen on the anterior side of the left chest, flexor aspect of left upper limb and palm.

**Figure 2:**
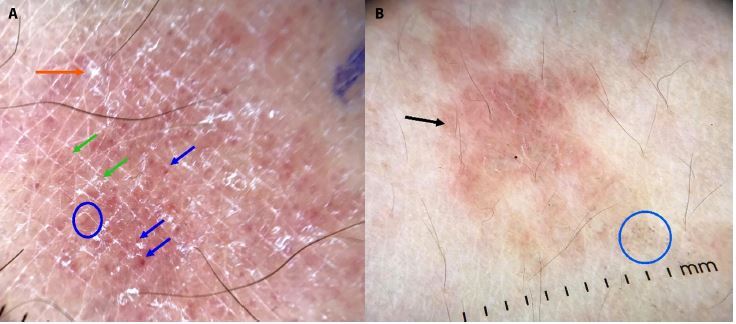
Dermoscopy showing red globules (black arrow), red (red arrow) and brown dots (blue circle), scaling (blue arrow), linear white (green arrow) and red lines (blue circle) with coppery-red background (DermLite DL4, ×10, Polarized light).

**Figure 3:**
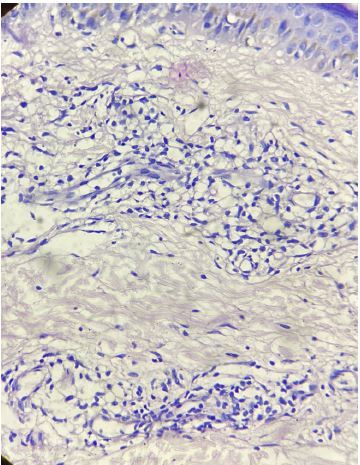
Histopathological analysis showed mildly atrophic epidermis with basal layer displaying focal vacuolization and dense perivascular lymphocytic infiltrate in the dermis.

A 32-year-old male presented with mildly itchy erythematous rash covering the left side of the chest, arm and forearm. The rash was ongoing in the last 3–4 months. On cutaneous examination, there were multiple erythematous, slightly scaly round-to-oval patches, varying from 0.5–5 cm in size, associated with coppery tinge, seen on the anterior part of left chest (not crossing the midline), flexor aspect of left upper limb and left palm (Figure 1). Other cutaneous and systemic examinations were normal. Routine investigations were within normal ranges. Differential diagnosis of ULC and linear PR were considered. Dermoscopy was performed using DermLite IV at 10× magnification, and revealed coppery-red background, linear white and red lines, red globules, red and brown dots, and scaling (Figure 2). On histopathological examination, the epidermis appeared mildly atrophic with loss of dermal papillae and the basal layer showed focal vacuolization (Figure 3). Dermis revealed edema, dense perivascular lymphocytic infiltrate, and extravasation of red blood cells (RBC). Based on these findings, diagnosis of ULC was made. The patient was treated conservatively, and he recovered within 2 weeks.

## Discussion

Dermoscopy can be used for differentiating ULC from linear PR. On dermoscopy of PPD, coppery-red background due to lymphohistiocytic dermal infiltration, extravasated RBCs, and hemosiderin deposition is observed. Red dots and globules represent extravasated RBCs and dilated blood vessels. Brown dots represent melanocytes in basal layer of epidermis and dermal melanophages in upper dermis. Scaling observed in our patient, might be due to the chronicity of the lesions. In PR, peripheral white scales and few red dots are seen in yellowish background [1].

Based on the clinical and dermoscopic findings we concluded that dermoscopic features of ULC are similar to the ones reported in PPD. In our case, scaling was an additional finding. Recently, a case report described dermoscopy of ULC. The authors observed dermoscopic features such as linear vessels, brown reticular lines, red dots, and clods with a brown-pigmented network [2]. In addition to these features, we also observed brown dots and linear white lines along dermatoglyphics secondary to scaling. To best of the authors’ knowledge, the dermoscopic features such as brown dots and scaling in ULC have not been reported in the literature yet. Thus, our findings could further help dermatologists in the diagnosis of ULC and differentiating it from linear PR, which might preclude the need for invasive procedures such as skin biopsy for a benign and self-resolving condition.
